# The role of hospitals in providing nutritious and sustainable foods for human and planetary health

**DOI:** 10.1017/S1368980025100396

**Published:** 2025-06-02

**Authors:** Simone Pettigrew, Daisy Coyle, Stefanie Carino, Fraser Taylor, Annet Hoek

**Affiliations:** 1 The George Institute for Public Health, University of New South Wales, Sydney, Australia; 2 Climate and Health Alliance, Melbourne, Australia

**Keywords:** Food, Health services, Hospitals, Sustainability

The health system is a substantial contributor to global warming while also having responsibility for addressing the adverse effects of climate change on human health. The latter includes the human suffering arising from a broad range of adverse health consequences resulting from a warming planet, including those relating to infectious diseases; respiratory, cardiovascular and neurological outcomes and mental illness^(1)^. It is estimated that if the global health system was a country, it would be the fifth largest emitter of greenhouse gases^(2)^. The health system must move to net zero to stop being part of the problem, which will have co-benefits by positively impacting human health and mitigating climate change^(3–5)^. A more environmentally sustainable health system is possible, but it will take time and will require a comprehensive approach to implementation. For example, Kaiser Permanente Health Services in the United States achieved carbon neutrality in 2020 through their conversion to renewable energy sources but have set a 2050 target for net zero emissions in recognition of the time it will take to influence other parts of the supply chain for the enormous range of goods and services used in health services^(6)^.

The food system is also a major contributor to climate change, responsible for around one-third of global anthroprogenic greenhouse gas emissions^(7)^. Over half of these emissions (57 %) are attributed to red meat and dairy products^(7)^. Radical changes need to be made to food production and consumption as the world exceeds the Paris Agreement target of limiting global temperature rises to 1·5°C above pre-industrial temperatures^(8)^.

There is an inextricable link between the food system and both human and planetary health. In general, more nutritious diets are more environmentally sustainable and more sustainable diets are more nutritious^(9,10)^. It is estimated that a global transition to healthy, low-emission diets could prevent more than 12 million deaths per year^(7)^. In the meantime, the current unhealthy food system produces enormous negative externalities resulting from the food industry failing to absorb the full costs associated with the health and environmental consequences of its products^(11)^. Overall, the human and planetary harms imposed by the food system are valued at around $US15 trillion, which far exceeds the value the food system creates^(12)^. There is a critical need to restructure agricultural processes to deliver both healthier and more sustainable diets, which will require major changes all along the supply chain^(13)^. Additionally, influencing consumer choices towards more sustainable alternatives can reduce the carbon impact of the food system on the environment and accelerate industry efforts to improve the sustainability of their product offerings^(14)^.

## Food provision in health services

At the intersection of the health system and the food system are the food provision functions within health services. Currently, there are substantial negative environmental impacts across all phases of food provision within health services, including food procurement, preparation, consumption and waste management^(15)^. There is a recognised need for immediate action to adapt food provision within hospitals, for example, to ensure the meals provided are both nutritious and sustainable to optimise human and planetary health^(7,16)^. Previous research has found that up to 17 % of hospitals’ climate impact results from activities associated with food provision^(17)^.

As both key public health actors and major food providers in the community, hospitals should be a priority for sustainable food initiatives^(18,19)^. Such initiatives represent useful initial decarbonisation pathways, as changes in internal food policies and practices are more likely to be immediately feasible compared to attempting to modify aspects of clinical practice (e.g. re-using surgical equipment) that have patient safety implications^(17)^.

Two primary strategies discussed in the literature for improving the sustainability of hospital food provision are (i) incorporating more plant foods into meals and menus and (ii) reducing food waste. As well as reducing greenhouse emissions, both these strategies have co-benefits in the form of reducing water and land use and increasing biodiversity^(7,20)^. Recommended approaches for increasing plant food content include placing more vegetarian dishes on the menu, listing vegetarian choices first and reducing the quantity of meat served in mixed dishes^(17,19,21)^. Suggested methods of addressing food waste include decreasing portion sizes, timing meal selection as close to meal delivery as possible to prevent food being prepared for discharged patients and making untouched meals available to staff at discounted prices^(17,22)^.

Internal factors found to be critical for successful food sustainability initiatives in hospitals include strong management commitment and supportive leadership, development and implementation of internal protocols, allocation of dedicated personnel to relevant tasks, flexible procurement policies that facilitate access to local food sources, staff education and accessing learnings from other hospitals^(6,19,23,24)^. External facilitating factors include government requirements for more sustainable practices and public support for climate change initiatives^(23–25)^. Identified challenges associated with introducing more sustainable food systems within health services are entrenched vested interests throughout the food supply chain, a lack of policy frameworks, restrictions on food sourcing, cost, poor communication within the organisation, resistance to changing catering routines and patient preferences^(12,15,18,23)^. In addition, politically uncomfortable shifts in current agricultural subsidy systems are required to address broader issues relating to the availability and affordability of healthy and sustainable food products in the Australian food supply^(14)^.

## The Australian National Health and Climate Strategy

Reflecting the available evidence, the Australian Government’s National Health and Climate Strategy^(26)^ acknowledges the importance of food provision in health services and recommends efforts to introduce sustainable procurement systems, update menus to include more nutritious low-carbon foods and eliminate food waste. These important objectives may be difficult for hospitals to implement in practice without a clear system to identify nutritious low-carbon food options to facilitate embedding these products in procurement guidelines

A key issue will be the identification of more sustainable food products for integration into hospital menus. While the relative advantage of plant foods over meat and dairy products is well-known and can provide direction for menu design and food procurement, determining the environmental impact of products from other food categories is considerably more difficult. The recipes and ingredient lists that signal nutritional quality are unable to provide much-needed information about the environmental effects of specific food products.

The planned incorporation of sustainability criteria into the Australian Dietary Guidelines will be of assistance, but by necessity, the Guidelines are likely to focus on broad product categories rather than specific meals and food products. They also only represent voluntary guidance, and as such, may not constitute a compelling motive for food producers and wholesalers to make the disruptive changes to their processes that are required to provide hospitals with ready access to more sustainable food product options. Furthermore, included as an action in the National Health and Climate Strategy is a review of how sustainability is included in state and territory policies on food access, food availability and food procurement in hospitals^(26)^. Recent advances in estimating the environmental impact of foods have the potential to better inform food provision decisions in hospitals and other health services. Detailed life cycle assessments are being conducted to develop databases covering the foods available in the marketplace. In Australia, the newly developed ecoSwitch database provides greenhouse gas emission estimates for packaged foods, and the Eco-score database performs a similar function in France^(14)^. These emerging information sources can provide important guidance for food service managers and procurement professionals attempting to modify their organisations’ food offerings to become more planet-friendly.

To conclude, there is a pressing need for climate action through systems-level interventions, including the implementation of sustainable food procurement processes in hospitals. This approach has the potential to provide beneficial health outcomes for patients, staff and visitors, while also making a meaningful contribution to the environmental sustainability of this critical sector. It will additionally provide an exemplar for other institutions (e.g. aged care services and schools) to reduce their ecological impact through modification of their food provision functions.
